# Bacterial Superantigen-Mediated Toxic Shock: Hyperinflammatory Trajectories and Extracorporeal Immunomodulation in Streptococcal Toxic Shock Syndrome

**DOI:** 10.3390/toxins18090388

**Published:** 2026-09-08

**Authors:** Kuo-Hao Lo, Kuo-Cheng Lu, Yen-Hung Liu, Yi-Chou Hou

**Affiliations:** 1 Department of Internal Medicine, Cardinal Tien Hospital, New Taipei City 231484, Taiwan; u101001801@cmu.edu.tw (K.-H.L.); 091668@mail.fju.edu.tw (Y.-H.L.); 2 Division of Nephrology, Department of Internal Medicine, Taipei Tzu Chi Hospital, Buddhist Tzu Chi Medical Foundation, New Taipei City 231405, Taiwan; kuochenglu@gmail.com; 3 Division of Nephrology, Department of Medicine, Fu Jen Catholic University Hospital, School of Medicine, Fu Jen Catholic University, New Taipei City 24352, Taiwan; 4 School of Medicine, College of Medicine, Fu Jen Catholic University, New Taipei City 242062, Taiwan

**Keywords:** streptococcal toxic shock syndrome, group A *Streptococcus*, streptococcal superantigens, hemoadsorption, extracorporeal immunomodulation, hyperinflammatory phenotype

## Abstract

Streptococcal toxic shock syndrome (STSS) is a fulminant manifestation of invasive group A *Streptococcus* infection characterized by superantigen-driven hyperinflammation, refractory shock, and rapidly progressive multiorgan dysfunction. Despite antimicrobial therapy, source control, intravenous immunoglobulin, and intensive organ support, some patients deteriorate rapidly, prompting interest in extracorporeal immunomodulation. This narrative review examines the biological rationale and clinical experience of hemoadsorption in STSS, supported by an illustrative case and comparison with published reports. Our patient developed vasopressor-dependent shock, acute kidney injury, thrombocytopenia, hepatic and myocardial injury, hyperlactatemia, and metabolic acidosis within 48 h. CytoSorb was integrated with continuous venovenous hemofiltration during ongoing deterioration, followed by hemodynamic and organ recovery; however, concurrent therapies preclude attribution of benefit to hemoadsorption. Published cases show a similar pattern, with extracorporeal escalation initiated during refractory shock and accumulating organ dysfunction rather than at a predefined biomarker threshold. Although experimental and observational evidence supports the biological activity of hemoadsorption, clinical evidence remains insufficient to establish a survival benefit. Current evidence does not support routine hemoadsorption for STSS. A rapidly progressive, multidimensional hyperinflammatory trajectory may instead provide a framework for future studies of patient selection and treatment timing.

## 1. Introduction

Streptococcal toxic shock syndrome (STSS) is a fulminant manifestation of invasive group A *Streptococcus* (GAS; *Streptococcus pyogenes*) infection, characterized by rapidly progressive shock and multiorgan dysfunction. Despite advances in antimicrobial therapy, source control, hemodynamic resuscitation, and organ support, mortality remains substantial [1,2]. A central pathogenic feature is toxin-mediated immune dysregulation: streptococcal pyrogenic exotoxins and related virulence factors act as superantigens, activating large populations of T lymphocytes through major histocompatibility complex class II molecules and selected Vβ regions of the T-cell receptor, resulting in extensive inflammatory mediator release [3]. This mechanism contributes to endothelial activation, vascular leakage, vasoplegia, microcirculatory dysfunction, coagulation abnormalities, and tissue injury, providing a biological explanation for the abrupt transition from localized infection to circulatory collapse.

Clinically, STSS usually presents as a multidimensional deterioration rather than isolated elevation of a single biomarker. Patients may develop escalating vasopressor requirements, hyperlactatemia, acute kidney injury (AKI), thrombocytopenia, hepatic dysfunction, myocardial depression, respiratory failure, and rapidly progressive soft-tissue injury over hours to days. Standard treatment therefore focuses on infection eradication and interruption of toxin production, including β-lactam therapy, clindamycin, rapid source control, organ support, and, in selected severe cases, intravenous immunoglobulin (IVIG). IVIG may neutralize streptococcal toxins and superantigens, but supporting evidence remains limited and largely observational [2,4].

Persistent vasoplegia and rapidly accumulating organ dysfunction despite conventional therapy have prompted interest in extracorporeal blood purification as adjunctive immunomodulation. CytoSorb hemoadsorption can reduce circulating concentrations of several inflammatory cytokines in experimental human settings [5], while clinical studies in septic shock have shown variable effects on vasopressor requirements, inflammatory markers, and outcomes [6,7,8]. Consequently, routine hemoadsorption is not supported, and its role in STSS remains undefined.

This uncertainty shifts attention from whether an adsorption device is intrinsically effective to which patients might represent a biologically plausible target and when treatment should be considered. The Acute Dialysis Quality Initiative (ADQI) framework emphasizes sepsis heterogeneity and supports phenotype-based selection linked to plausible biological targets rather than indiscriminate device use [9]. STSS may be a relevant model because superantigen-mediated immune activation is central to its pathogenesis. In this narrative review, we use an illustrative case of fulminant *S. pyogenes* STSS and published cases to examine the trajectory preceding extracorporeal treatment, focusing on hyperinflammatory phenotype recognition, organ dysfunction, timing, and patient selection.

## 2. Illustrative Case and Literature Comparison

### 2.1. Illustrative Clinical Case

**Figure 1:**
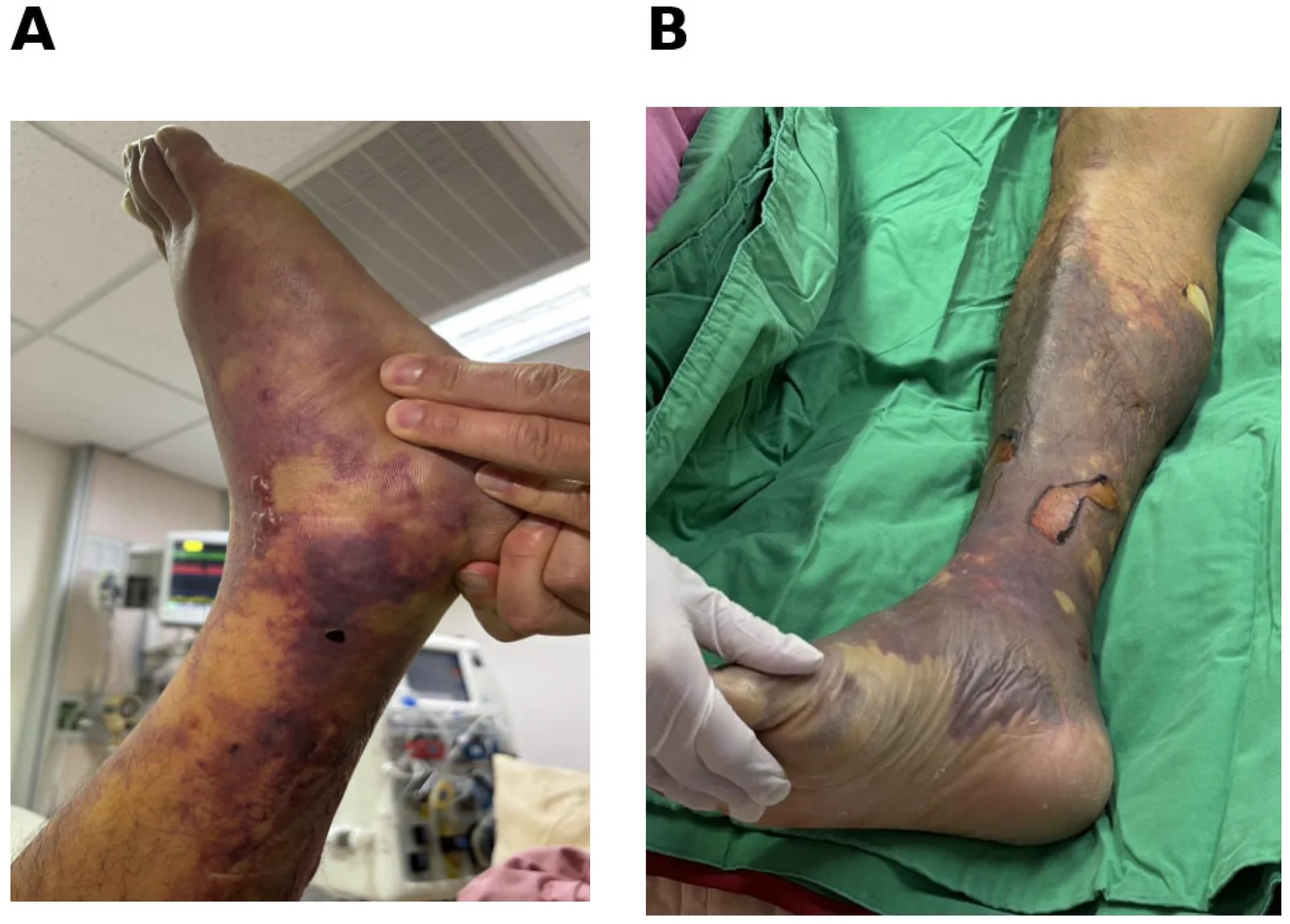
Rapid progression of soft-tissue manifestations in streptococcal toxic shock syndrome. (**A**) Right lower extremity on admission, showing extensive erythema, swelling, ecchymotic discoloration, and hemorrhagic bullae associated with invasive *Streptococcus pyogenes* infection. (**B**) Progression 24 h after admission, showing worsening bullous lesions, ischemic discoloration, and extensive soft-tissue involvement, paralleling rapid systemic deterioration and evolving multiorgan dysfunction.

A previously healthy 44-year-old man presented after 2 days of fever, chills, severe pain, and rapidly progressive swelling of the right lower extremity. On admission, he was febrile, hypotensive, tachycardic, and tachypneic. Examination showed diffuse erythema, marked edema, ecchymotic discoloration, and hemorrhagic bullae, which progressed within 24–48 h to ischemic discoloration, bullous change, and soft-tissue necrosis. Blood cultures subsequently grew *Streptococcus pyogenes*, establishing invasive GAS infection consistent with STSS (Box 1, Figure 1).

Box 1 **Illustrative Observation (independent case).**

**The single-patient observation in Section 2.1 is presented separately from the review argument as an illustrative example of the rapidly progressive, multidimensional hyperinflammatory phenotype discussed in this review; it is not offered as evidence of treatment efficacy.**

During the first 48 h, laboratory and clinical abnormalities progressed simultaneously. WBC increased from 8.79 to 27.25 × 10^3^/μL, platelet count fell from 254 to 82 × 10^3^/μL, serum creatinine increased from 2.05 to 6.59 mg/dL, and lactate rose from 4.64 to 7.95 mmol/L. Concurrent hyperbilirubinemia, transaminase elevation, myocardial injury, hyperkalemia, and persistent metabolic acidosis indicated evolving multiorgan dysfunction syndrome (MODS) (Table 1).

Despite broad-spectrum antimicrobial therapy, fluid resuscitation, and escalating vasopressor support, the patient developed vasopressor-dependent septic shock, respiratory failure requiring mechanical ventilation, severe AKI, refractory metabolic acidosis with hyperkalemia, thrombocytopenia, hepatic dysfunction, myocardial injury, and multiorgan dysfunction within approximately 48 h. IVIG was administered, and continuous venovenous hemofiltration (CVVH) was initiated for severe AKI and metabolic derangements. Because shock and multiorgan dysfunction continued to progress despite antimicrobial therapy, IVIG, hemodynamic support, and CVVH, CytoSorb hemoadsorption was incorporated into the CVVH circuit on hospital day 2. The decision was based on the aggregate trajectory of rapidly progressive multiorgan dysfunction, not a predefined cytokine threshold.

Three consecutive CytoSorb cartridges were used. Vasopressor requirements subsequently decreased, metabolic acidosis and hyperkalemia resolved, urine output recovered, and organ function improved. Mechanical ventilation and kidney replacement therapy were discontinued. The patient required prolonged treatment for soft-tissue injury, osteomyelitis, repeated debridement, wound care, and rehabilitation but survived to hospital discharge. Because multiple therapies were administered simultaneously, the independent contribution of hemoadsorption cannot be determined. This case is therefore used not as evidence of efficacy, but as an example of the rapidly progressive multidimensional phenotype that has prompted extracorporeal immunomodulation in STSS.

Temporal course of the extracorporeal intervention. Following integration of CytoSorb into the CVVH circuit during the night of hospital day 2, each cartridge was exchanged approximately every 24 h, and three consecutive cartridges were used over approximately 72 h of continuous hemoadsorption. At initiation, noradrenaline was administered at approximately 0.61 µg/kg/min, together with vasopressin. Although the vasopressor requirement subsequently decreased, sufficiently granular dose-by-time records were unavailable to determine the precise timing and magnitude of the initial reduction; hemodynamic stabilization permitting discontinuation of CVVH was documented by approximately 156 h after initiation. Lactate first decreased to <2 mmol/L (1.97 mmol/L) by approximately 48–60 h, although subsequent values fluctuated. Hyperkalemia resolved within approximately 24 h, arterial pH normalized by approximately 60 h, and the metabolic component of the acidosis substantially improved by approximately 108 h. CVVH was discontinued on October 29 and temporarily replaced by intermittent hemodialysis. Dialysis was no longer required after November 12, when a urine output of 3840 mL/day without furosemide was documented, approximately 480 h after CytoSorb initiation. These intervals describe temporal associations only and cannot establish causation, given the concurrent antimicrobial therapy, IVIG, source-control procedures, resuscitation, and CVVH.

### 2.2. Published Clinical Experience

Published experience remains extremely limited. Four previously reported cases of severe streptococcal or related toxin-mediated toxic shock treated with extracorporeal adsorption or related strategies were identified and compared with the present case: Hetz et al. 2014, Berkes et al. 2017, Amerson et al. 2024, and Miranda et al. 2025 [10,11,12,13]. Despite heterogeneity in age, pathogen documentation, source, extracorporeal modality, and timing, all reports shared severe circulatory failure with rapidly evolving multiorgan dysfunction before extracorporeal escalation (Table 2).

Hetz et al. described GAS necrotizing fasciitis with catecholamine-refractory shock, MODS, capillary leak, lactate 8.22 mmol/L, acidosis, mechanical ventilation, vasopressors, CRRT, and CytoSorb, with survival [10]. Berkes et al. reported a 5-year-old girl with toxin-mediated shock, profound acidosis, DIC, elevated lactate, and renal dysfunction; CytoSorb was combined with continuous venovenous hemodialysis approximately 78 h after ICU admission, and the patient survived [11]. Amerson et al. described confirmed *S. pyogenes* STSS with refractory vasoplegia despite VA-ECMO, IVIG, and renal replacement therapy; sequential extracorporeal therapy was added, and the patient survived [12]. Miranda et al. reported GAS bacteremia with vasoplegic shock, thrombocytopenia, KDIGO stage 3 AKI, hyperbilirubinemia, MODS, mechanical ventilation, and continuous venovenous hemodiafiltration with adjunctive hemoadsorption initiated on hospital day 2; the patient survived [13].

## 3. Discussion

STSS is a distinctive infection-associated shock syndrome in which invasive GAS virulence and an intense host immune response converge rapidly. The present case illustrates this trajectory: localized soft-tissue infection progressed within approximately 48 h to vasopressor-dependent shock, respiratory failure, severe AKI requiring CRRT, thrombocytopenia, hepatic injury, myocardial dysfunction, and MODS. The key observation is not that hemoadsorption caused recovery, but that this rapid multidimensional deterioration represents the clinical context in which extracorporeal immunomodulation has been considered [1,14].

*Streptococcus pyogenes* produces virulence factors, including streptococcal pyrogenic exotoxins, that can function as superantigens. By activating large T-cell populations through MHC class II molecules and selected Vβ regions of the T-cell receptor, these toxins can trigger extensive cytokine release and downstream endothelial dysfunction, vasodilatation, capillary leak, microcirculatory injury, coagulopathy, and tissue damage [3]. In the present case, rising leukocyte count, falling platelets, worsening creatinine, increasing lactate, hyperbilirubinemia, metabolic acidosis, and myocardial dysfunction evolved concurrently. Their significance lies less in any single threshold than in their velocity and multisystem concurrence.

This concept is particularly relevant to our patient. During the first 48 h, the leukocyte count increased from 8.79 to 27.25 ×10^3^/μL, platelet count fell from 254 to 82 ×10^3^/μL, serum creatinine increased from 2.05 to 6.59 mg/dL, lactate rose from 4.64 to 7.95 mmol/L, and total bilirubin increased from 1.20 to 4.85 mg/dL. These abnormalities evolved concurrently with worsening vasopressor dependence, respiratory failure, metabolic acidosis, and newly developed myocardial dysfunction. Rather than any single laboratory threshold, it is the simultaneous, rapidly accelerating involvement of hemodynamic, renal, hematologic, hepatic, and myocardial domains that defines this phenotype; this concurrence and velocity—not the absolute value of any one marker—plausibly identify the narrow window in which adjunctive immunomodulation could be studied before injury becomes irreversible.

### 3.1. Clinical Trajectory May Be More Informative than a Single Biomarker

No validated cytokine, inflammatory biomarker, lactate, or organ-failure threshold currently identifies STSS patients who should receive hemoadsorption. The broader sepsis literature highlights this problem: Acute Dialysis Quality Initiative (ADQI) emphasized that extracorporeal blood purification should be studied in biologically plausible phenotypes rather than applied indiscriminately to heterogeneous septic shock populations [9]. In the limited STSS reports reviewed here, escalation generally occurred after persistent vasopressor-dependent shock, mechanical ventilation, AKI or CRRT, metabolic acidosis, hyperlactatemia, thrombocytopenia or coagulopathy, and progressive MODS had emerged. These findings suggest a recurring clinical trajectory, not a validated indication.

Attempting an answer from the five-case pool (hypothesis, not a validated indication). Cross-referencing all five patients by the metrics most consistently reported—time to initiation, baseline vasopressor requirement, baseline lactate, and time to hemodynamic stabilization—reveals a convergent pattern despite heterogeneity. Extracorporeal escalation clustered early, from approximately 6 h to 3 days after ICU admission or after preceding rescue therapy (Hetz et al. after progression to catecholamine-refractory shock [10]; Berkes et al. approximately 78 h after ICU admission [11]; Amerson et al. approximately 6 h after ECMO/IVIG [12]; Miranda et al. and the present case on hospital day 2 [13]). Where reported, baseline lactate was high (Hetz 8.22 mmol/L; Berkes 7 mmol/L; present case rising from 4.64 to 7.95 mmol/L), and all patients required escalating vasopressors and continuous renal replacement therapy. Baseline vasopressor dose and time to hemodynamic stabilization were inconsistently reported in the published cases. In the present case, noradrenaline was administered at approximately 0.61 µg/kg/min together with vasopressin at CytoSorb initiation; sufficiently granular dose-by-time data were unavailable to determine the precise timing of the initial vasopressor reduction, although hemodynamic stabilization permitting discontinuation of CVVH was documented approximately 156 h after initiation. This reporting inconsistency underscores the need for standardized prospective data capture. On this basis, we propose, explicitly as a hypothesis generated from a five-case pool, a preliminary optimal-candidate framework: (i) confirmed or strongly suspected invasive group A streptococcal infection with a biologically plausible superantigen-mediated mechanism [15]; (ii) objective, rapid multi-domain deterioration despite adequate source control, antimicrobial therapy, IVIG, and resuscitation [16,17]—escalating vasopressor dose, non-clearing lactate, new acute kidney injury requiring CRRT, thrombocytopenia or coagulopathy, and hepatic or myocardial injury [18,19]; and (iii) initiation before established, irreversible MODS. We frame this as a clinical algorithm to be tested prospectively rather than a treatment recommendation.

The 2024 Asia-Pacific oXiris Expert Meeting consensus provides a useful comparator because it explicitly frames extracorporeal blood purification as a therapy for selected patients with sepsis or hyperinflammatory conditions rather than as routine supportive care. The consensus describes oXiris as a multifunctional CRRT membrane combining hemofiltration, endotoxin adsorption, cytokine adsorption, and a heparin-grafted surface intended to reduce thrombogenicity, features supported by clinical crossover data showing greater reductions in endotoxin and selected cytokines with oXiris than with a standard CRRT filter in septic shock-associated AKI [14,20]. Importantly, most consensus statements were formulated as recommendations or practice points with low-to-moderate certainty rather than definitive evidence of outcome benefit [20]. This is highly relevant to STSS: although GAS STSS is not primarily endotoxin-driven, the consensus reinforces the broader principle that adsorption therapies should be considered only when the clinical phenotype suggests uncontrolled inflammatory mediator activity, hemodynamic instability, AKI requiring CRRT, severe lactic acidosis, or coagulation disturbance.

### 3.2. Biological Rationale for Hemoadsorption

CytoSorb is a porous polymer hemoadsorption system intended to reduce circulating hydrophobic molecules within an intermediate-molecular-weight range. Its rationale in STSS is not direct elimination of GAS or specific neutralization of streptococcal superantigens, but modulation of the downstream inflammatory cascade. By contrast, oXiris integrates adsorption into a CRRT membrane and includes endotoxin-adsorptive and cytokine-adsorptive properties in addition to kidney support; in a randomized crossover double-blind study, oXiris reduced endotoxin and several cytokines more effectively than a standard filter during CRRT for septic shock-associated AKI [14]. This distinction matters because results from one device cannot be automatically extrapolated to another. Human endotoxemia data show that CytoSorb can attenuate circulating cytokines such as TNF, IL-6, IL-8, and IL-10 [5]; however, experimental endotoxemia differs substantially from invasive GAS infection with established shock and MODS. Thus, biological plausibility should not be equated with clinical efficacy.

Clinical evidence for CytoSorb remains mixed. Observational studies have reported improved hemodynamics or lower-than-expected mortality in selected septic shock cohorts [6], but such analyses are vulnerable to indication bias, survivor bias, confounding, and institutional practice effects. Randomized studies have been less convincing: one trial in severe sepsis/septic shock with ARDS showed IL-6 removal without improved outcomes [7], and the REMOVE trial in infective endocarditis surgery found no improvement in postoperative organ dysfunction [21]. A recent meta-analysis reported associations with lower short-term mortality and reduced vasopressor requirements, but emphasized heterogeneity and modest study quality [8]. Therefore, routine hemoadsorption cannot be recommended for STSS, and the present case cannot establish causality.

Cost and resource implications. Cost and resource use are non-trivial and represent a real barrier to both routine adoption and trial feasibility. In the present case, three consecutive CytoSorb cartridges were required in addition to the CRRT circuit, intensified ICU nursing, and monitoring. Based on local institutional procurement, the estimated acquisition cost was approximately USD 3500–4000 per cartridge, corresponding to an incremental device cost of approximately USD 10,500–12,000 for the complete three-cartridge hemoadsorption course over CRRT alone. These estimates are approximate and may vary across institutions, procurement agreements, jurisdictions, and exchange rates. Available economic evaluations of hemoadsorption are limited, derive largely from other indications, and reach heterogeneous conclusions [22]; in the absence of a demonstrated survival or morbidity benefit, cost cannot be assumed to be offset. These considerations reinforce that hemoadsorption should not be applied indiscriminately and that future trials must incorporate predefined health-economic endpoints.

If hemoadsorption has a role, timing is likely inseparable from phenotype. Treatment too early may offer little benefit if the inflammatory burden is limited, whereas treatment after irreversible microcirculatory, mitochondrial, or cellular injury may be futile. The biologically plausible window may lie during rapid refractory deterioration, before irreversible MODS is established. In this case and in published STSS reports, extracorporeal escalation clustered early during worsening shock and accumulating organ dysfunction, supporting future investigation of trajectory-based enrollment criteria rather than broad sepsis definitions [23,24].

The oXiris consensus also emphasizes a practical treatment window. It suggests that, when oXiris is used in septic or hyperinflammatory patients requiring CRRT, earlier initiation may be favored in those with persistent hemodynamic instability after adequate resuscitation, high vasopressor dose, severe lactic acidosis, worsening SOFA or APACHE II scores, or elevated inflammatory or endotoxin-related biomarkers [15]. This approach is consistent with published oXiris clinical experience in abdominal septic shock, where early oXiris-based CRRT was associated with reductions in lactate, procalcitonin, SOFA score, and vasopressor requirement, although evidence remains limited to uncontrolled clinical observations [25]. Although these criteria were developed for oXiris and not CytoSorb, they support the same conceptual direction proposed in this review: patient trajectory should drive consideration of extracorporeal immunomodulation. In STSS, a comparable decision framework would not rely on endotoxin levels but on the rapid convergence of vasoplegia, lactate kinetics, AKI, thrombocytopenia, hepatic dysfunction, myocardial depression, and escalating organ support.

Monitoring and discontinuation are equally important. The oXiris consensus highlights hemodynamic response, lactate reduction, Sequential Organ Failure Assessment (SOFA) evolution, oxygenation, urine output, and inflammatory markers during the first 24–72 h as key variables for evaluating response, and it cautions against continuing therapy when no clinical improvement is observed [21]. The emphasis on norepinephrine dose, lactate, inflammatory biomarkers, and SOFA score is consistent with the outcome domains used in oXiris trials and meta-analyses, although overall certainty remains very low and randomized evidence has not confirmed a robust mortality benefit [20,26]. This is directly applicable to future STSS research. If hemoadsorption is investigated, treatment protocols should predefine early response domains, including vasopressor dose reduction, lactate clearance, renal recovery or CRRT dependence, platelet trajectory, bilirubin, cardiac function, and need for ongoing mechanical ventilation.

STSS may be a more biologically coherent population for future extracorporeal immunomodulation studies than undifferentiated sepsis because superantigen-mediated immune activation is central to its pathogenesis. Nevertheless, STSS remains heterogeneous: age, bacterial strain, toxin repertoire, infection source, bacterial burden, source-control timing, and host immunity may all influence risk and response. Diagnosis alone should therefore not trigger hemoadsorption. A more rational research phenotype would combine confirmed or strongly suspected invasive GAS infection with rapidly increasing vasopressor needs, persistent hyperlactatemia, worsening AKI, thrombocytopenia or coagulopathy, hepatic injury, myocardial dysfunction, or rising organ-failure scores despite appropriate therapy [2,9].

### 3.3. Safety, Drug Adsorption, and Interaction with CRRT

Hemoadsorption should be regarded as a pharmacologically active extracorporeal therapy rather than simply an extension of CRRT. The adsorption spectrum is not limited to inflammatory mediators.

A recent systematic review of drug removal by CytoSorb evaluated available in vitro and in vivo evidence and demonstrated clinically relevant adsorption for selected antimicrobial and non-antimicrobial drugs, emphasizing the need for careful dose adjustment and therapeutic drug monitoring when feasible [27].

This consideration is particularly important in STSS, in which adequate antimicrobial exposure is essential. Any theoretical benefit from inflammatory mediator removal would be undermined if antibiotic concentrations became subtherapeutic.

Drug dosing must therefore account simultaneously for expanded volume of distribution in septic shock, residual kidney function, CRRT clearance, and extracorporeal adsorption. Where available, therapeutic drug monitoring may be especially useful for drugs with known or suspected adsorption.

The ability to integrate CytoSorb into an existing CRRT or extracorporeal membrane oxygenation (ECMO) circuit offers logistical convenience, but the presence of an extracorporeal circuit should not itself constitute an indication for hemoadsorption. Circuit availability reduces procedural complexity only after an independent clinical rationale for extracorporeal immunomodulation has been established.

The Asia-Pacific oXiris consensus further illustrates how extracorporeal blood purification should be operationalized: modality selection, delivered dose, circuit lifespan, anticoagulation strategy, and combination with ECMO or other extracorporeal therapies should be specified rather than left to ad hoc bedside practice [20]. These operational details matter in STSS because shock, coagulopathy, severe acidosis, and soft-tissue necrosis may simultaneously increase the need for CRRT and shorten filter life. Future studies should therefore report not only whether adsorption was used, but also cartridge duration, blood-flow rate, effluent dose, anticoagulation, interruptions, circuit clotting, antimicrobial dosing, and timing relative to debridement, IVIG, and vasopressor escalation.

### 3.4. A Proposed Clinical Interpretation: Trajectory Rather than Threshold

Taken together, our case and the limited STSS literature support a conceptual shift from threshold-based toward trajectory-based assessment.

We do not propose a numerical trigger for CytoSorb initiation. Instead, three domains may be considered as a research framework.

First, the underlying disease should have a biologically plausible hyperinflammatory mechanism, as occurs in superantigen-mediated STSS [3]. Second, there should be objective evidence of rapid progression despite appropriate conventional treatment, demonstrated by increasing vasopressor requirements, worsening tissue hypoperfusion, and accumulating organ dysfunction. The importance of early recognition and multidisciplinary treatment in STSS has been emphasized in contemporary ICU reviews [1]. Third, if extracorporeal immunomodulation is considered, it should remain adjunctive to source control, appropriate antimicrobial therapy, IVIG when clinically appropriate, and comprehensive organ support. This principle is consistent with phenotype-based extracorporeal blood purification concepts rather than device-driven treatment [9].

The central research question should therefore move away from “Does CytoSorb work in sepsis?” toward “Which biological phenotype, treated at which stage of deterioration and with what intensity, might derive benefit from extracorporeal immunomodulation?”.

This is particularly relevant to STSS, where rapid superantigen-mediated immune activation provides a plausible biological target but definitive therapeutic evidence remains absent.

Trajectory versus threshold: strengths, limitations, and the objection that this merely restates practice. A reasonable objection is that experienced clinicians already escalate therapy in response to aggregate deterioration rather than to a single biomarker, so a trajectory-based description may appear only to restate ordinary practice. We agree that the underlying clinical intuition is not novel. What is potentially useful, and currently absent, is the translation of that implicit judgment into prospectively defined, operationalized, and reproducible criteria—specific velocity thresholds across vasopressor dose, lactate, and renal, hematologic, hepatic, and cardiac domains—that could standardize enrollment and treatment timing in a trial [19,21]. The limitations are equally important: trajectory criteria risk circularity (sicker patients receive more therapy and are compared with themselves), remain non-specific to STSS, are vulnerable to lead-time and survivor bias, lack a discrete actionable trigger, and impose a measurement burden. We therefore present the framework strictly as hypothesis-generating.

### 3.5. Limitations and Future Directions

Several limitations must be acknowledged. First, our clinical observation involves a single patient and cannot establish causality. Second, all published STSS cases identified in our focused literature review received multiple simultaneous interventions, making the independent effect of hemoadsorption impossible to determine.

Third, favorable outcomes are more likely to be published, creating substantial publication bias. Fourth, serial cytokine and superantigen measurements were unavailable in our patient; therefore, the proposed hyperinflammatory phenotype was inferred from the clinical trajectory rather than demonstrated by molecular profiling.

Fifth, extracorporeal blood-purification techniques are not interchangeable. Results obtained with CytoSorb should not automatically be extrapolated to polymyxin-B hemoperfusion, oXiris, plasma exchange, or other adsorption systems.

Future studies should therefore move beyond uncontrolled rescue reports. A prospective STSS registry should capture vasopressor dose trajectories, lactate kinetics, SOFA components, platelet counts, creatinine and urine output, bilirubin, cardiac function, CRRT and ECMO timing, source-control timing, antimicrobial exposure, IVIG use, and serial inflammatory biomarkers.

Beyond survival, the registry should capture morbidity-oriented outcomes—limb salvage versus amputation, kidney recovery and dialysis independence, myocardial recovery, and validated functional and health-related quality-of-life status at discharge and follow-up—because survival with major disability is a substantially different outcome, and long-term impairment after necrotizing soft-tissue infection is common [28,29,30].

Such data may determine whether a reproducible clinical trajectory precedes irreversible MODS and whether this trajectory identifies a biologically coherent population suitable for future controlled trials.

The oXiris consensus is also a reminder that consensus statements can guide practice but should not substitute for controlled evidence. Many oXiris recommendations are supported by observational data, small randomized studies, real-world cohorts, and expert agreement rather than large definitive trials [20]. A recent systematic review and meta-analysis found that oXiris use in critically ill patients was associated with lower overall mortality, reduced norepinephrine dose, lower lactate and IL-6 concentrations, and improved organ function, but all included studies had important risk of bias and the certainty of evidence was very low [26]. For STSS, this evidentiary gap is even larger. A pragmatic research pathway could begin with a multicenter registry using standardized trajectory definitions and then progress to adaptive trials enrolling patients early after fulfillment of prespecified hyperinflammatory and organ-support criteria.

In conclusion, STSS provides a clinically compelling but unproven setting for extracorporeal immunomodulation. Superantigen-driven biology provides a mechanistic rationale for targeting excessive host inflammation [3], while experimental human data confirm that CytoSorb can attenuate circulating inflammatory cytokines [5]. However, randomized and controlled clinical evidence remains insufficient to support routine hemoadsorption [7,8,21].

The principal lesson from our case and the available literature is therefore not that CytoSorb is an established treatment for STSS, but that early recognition of a rapidly progressive, multidimensional hyperinflammatory trajectory may provide a rational framework for identifying patients in whom adjunctive extracorporeal immunomodulation could be studied. Patient phenotype, velocity of deterioration, timing, and integration with established STSS management may ultimately be more important than the adsorption device itself.

## 4. Conclusions

STSS is a rapidly progressive, toxin-mediated syndrome in which superantigen-driven immune activation can lead to refractory shock and multiorgan dysfunction within a narrow therapeutic window. Although hemoadsorption provides a biologically plausible approach to modulating excessive host inflammation, current evidence remains insufficient to support its routine use or establish a survival benefit in STSS. The available clinical reports, including our illustrative case, suggest that extracorporeal escalation has generally been considered during rapidly worsening shock with concurrent metabolic, renal, hematologic, hepatic, and cardiovascular dysfunction rather than at a predefined cytokine or biomarker threshold. These observations support a shift from threshold-based to trajectory-based assessment, integrating disease phenotype, velocity of organ deterioration, and treatment timing. Extracorporeal immunomodulation should remain adjunctive to prompt antimicrobial therapy, toxin-suppressive strategies, source control, IVIG when appropriate, and comprehensive organ support. Future prospective studies should determine whether a reproducible hyperinflammatory trajectory can identify a biologically coherent subgroup of patients most likely to benefit from extracorporeal immunomodulation and define the optimal therapeutic window.

## Figures and Tables

**Table 1:** Clinical laboratory trajectory during the first 48 h of streptococcal toxic shock syndrome.

Parameter	Reference	Day 0 (10/20)	Day 1 (10/21)	Day 2 (10/22)
WBC (×10^3^/μL)	4–10	8.79	19.41	27.25
Neutrophils (%)	40–75	79.0	38.5	91.3
Band forms (%)	<5	8.0	58.7	2.9
Platelet (×10^3^/μL)	150–400	254	105	82
CRP (mg/dL)	<0.5	13.75	39.22	26.63
Creatinine (mg/dL)	0.7–1.3	2.05	5.80	6.59
eGFR (mL/min/1.73 m^2^)	>60	38.8	11.1	9.5
BUN (mg/dL)	7–20	26	69	74
Potassium (mmol/L)	3.5–5.0	4.37	5.18	5.54
AST (U/L)	<40	343	138	679
ALT (U/L)	<40	314	397	413
Total bilirubin (mg/dL)	<1.2	1.20	2.18	4.85
Troponin-I (ng/mL)	<0.04	0.0119	20.04	22.04
CK-MB (ng/mL)	<5	0.9	85	95.3
CK (U/L)	40–200	94	1222	1417
Arterial pH	7.35–7.45	7.22	7.28	7.30
HCO_3_^−^ (mmol/L)	22–26	13.6	12.6	14.0
Lactate (mmol/L)	<2.0	4.64	5.04	7.95

Laboratory findings demonstrate rapid and concurrent progression of systemic inflammation, thrombocytopenia, acute kidney injury, hepatic dysfunction, myocardial injury, metabolic acidosis, and tissue hypoperfusion. CytoSorb-integrated continuous venovenous hemofiltration (CVVH) was initiated on hospital day 2. Abbreviations: ALT, alanine aminotransferase; AST, aspartate aminotransferase; BUN, blood urea nitrogen; CK, creatine kinase; CK-MB, creatine kinase-MB; CRP, C-reactive protein; CVVH, continuous venovenous hemofiltration; eGFR, estimated glomerular filtration rate; HCO_3_^−^, bicarbonate; WBC, white blood cell count.

**Table 2:** Published clinical experience with extracorporeal immunomodulation in streptococcal and related toxin-mediated toxic shock.

Author/Year	Age/Sex	Pathogen	Major Clinical Features Before Therapy	Key Laboratory Findings	Organ Support Before/with Hemoadsorption	Timing of Extracorporeal Therapy	Outcome
Hetz et al., 2014 [10]	60 y/o woman	Group A β-hemolytic *Streptococcus*	Necrotizing fasciitis, refractory septic shock, MODS, capillary leak syndrome	pH 7.26, BE −9.2 mmol/L, lactate 8.22 mmol/L	Mechanical ventilation, vasopressors, CRRT + CytoSorb	Initiated after progression to catecholamine-refractory shock and MODS	Survived
Berkes et al., 2017 [11]	5 y/o girl	Toxin-mediated TSS	Severe pediatric TSS with circulatory collapse and DIC	pH 6.77, HCO_3_ 5.2 mmol/L, lactate 7 mmol/L, CRP 260.98 mg/L, creatinine 223 μmol/L	ICU support, CVVHD + CytoSorb	CytoSorb initiated approximately 78 h after ICU admission	Survived
Amerson et al., 2024 [12]	28 y/o woman	*Streptococcus pyogenes*	Refractory STSS despite VA-ECMO, severe vasoplegia and MODS	Severe hyperinflammatory phenotype reported	VA-ECMO, IVIG, RRT, sequential extracorporeal therapy	Seraph initiated approximately 6 h after ECMO/IVIG	Survived
Miranda et al., 2025 [13]	70 y/o man	GAS bacteremia	Vasoplegic septic shock, thrombocytopenia, MODS	Progressive vasopressor escalation, KDIGO stage 3 AKI, hyperbilirubinemia	Mechanical ventilation, CVVHDF + hemoadsorption	Initiated on hospital day 2 because of persistent shock progression	Survived
Present case	44 y/o man	*Streptococcus pyogenes*	Bullous cellulitis, vasopressor-dependent septic shock, respiratory failure, AKI, shock liver, cardiac dysfunction (LVEF 38%), MODS	WBC peak 27.25 × 10^3^/μL, neutrophilia 91.3%, platelet nadir 82 × 10^3^/μL, severe metabolic acidosis with hyperkalemia	Mechanical ventilation, vasopressors, IVIG, CVVH + CytoSorb	CytoSorb integrated with CVVH initiated on hospital day 2 during rapidly progressive hyperinflammatory deterioration	Survived

Published cases were compared according to pathogen, major clinical phenotype, laboratory abnormalities, organ support, extracorporeal modality, treatment timing, and outcome. The report by Berkes et al. described toxin-mediated pediatric toxic shock syndrome rather than unequivocally culture-confirmed group A streptococcal STSS and was included as a mechanistically related comparator [11]. The available cases are descriptive and should not be interpreted as evidence of treatment efficacy. Abbreviations: AKI, acute kidney injury; BE, base excess; CRP, C-reactive protein; CRRT, continuous renal replacement therapy; CVVH, continuous venovenous hemofiltration; CVVHD, continuous venovenous hemodialysis; CVVHDF, continuous venovenous hemodiafiltration; DIC, disseminated intravascular coagulation; GAS, group A *Streptococcus*; IVIG, intravenous immunoglobulin; KDIGO, Kidney Disease: Improving Global Outcomes; LVEF, left ventricular ejection fraction; MODS, multiorgan dysfunction syndrome; RRT, renal replacement therapy; STSS, streptococcal toxic shock syndrome; TSS, toxic shock syndrome; VA-ECMO, venoarterial extracorporeal membrane oxygenation.

## Data Availability

The clinical data presented in this article are not publicly available due to patient privacy and ethical restrictions. Relevant de-identified data may be available from the corresponding author upon reasonable request and subject to institutional and ethical requirements.

## Abbreviations

Abbreviation	Full term
ADQI	Acute Dialysis Quality Initiative
AKI	Acute kidney injury
APACHE II	Acute Physiology and Chronic Health Evaluation II
ARDS	Acute respiratory distress syndrome
CKRT	Continuous kidney replacement therapy
CRRT	Continuous renal replacement therapy
CVVH	Continuous venovenous hemofiltration
DIC	Disseminated intravascular coagulation
ECMO	Extracorporeal membrane oxygenation
GAS	Group A *Streptococcus*
ICU	Intensive care unit
IL	Interleukin
IVIG	Intravenous immunoglobulin
KDIGO	Kidney Disease: Improving Global Outcomes
MHC	Major histocompatibility complex
MODS	Multiorgan dysfunction syndrome
PMID	PubMed identifier
SOFA	Sequential Organ Failure Assessment
STSS	Streptococcal toxic shock syndrome
TNF	Tumor necrosis factor
VA-ECMO	Venoarterial extracorporeal membrane oxygenation

## References

[1] Schmitz M., Roux X., Huttner B., Pugin J. (2018). Streptococcal toxic shock syndrome in the intensive care unit. Ann. Intensive Care.

[2] Bartoszko J.J., Elias Z., Rudziak P., Lo C.K.L., Thabane L., Mertz D., Loeb M. (2022). Prognostic factors for streptococcal toxic shock syndrome: Systematic review and meta-analysis. BMJ Open.

[3] McCormick J.K., Yarwood J.M., Schlievert P.M. (2001). Toxic shock syndrome and bacterial superantigens: An update. Annu. Rev. Microbiol..

[4] Parks T., Wilson C., Curtis N., Norrby-Teglund A., Sriskandan S. (2018). Polyspecific Intravenous Immunoglobulin in Clindamycin-treated Patients With Streptococcal Toxic Shock Syndrome: A Systematic Review and Meta-analysis. Clin. Infect. Dis..

[5] Jansen A., Waalders N.J.B., van Lier D.P.T., Kox M., Pickkers P. (2023). CytoSorb hemoperfusion markedly attenuates circulating cytokine concentrations during systemic inflammation in humans in vivo. Crit. Care.

[6] Brouwer W.P., Duran S., Kuijper M., Ince C. (2019). Hemoadsorption with CytoSorb shows a decreased observed versus expected 28-day all-cause mortality in ICU patients with septic shock: A propensity-score-weighted retrospective study. Crit. Care.

[7] Schädler D., Pausch C., Heise D., Meier-Hellmann A., Brederlau J., Weiler N., Marx G., Putensen C., Spies C., Jörres A. (2017). The effect of a novel extracorporeal cytokine hemoadsorption device on IL-6 elimination in septic patients: A randomized controlled trial. PLoS ONE.

[8] Steindl D., Schroeder T., Krannich A., Nee J. (2025). Hemoadsorption in the Management of Septic Shock: A Systematic Review and Meta-Analysis. J. Clin. Med..

[9] Kellum J.A., Gómez H., Gómez A., Murray P., Ronco C. (2016). Acute dialysis quality initiative (adqi) xiv sepsis phenotypes and targets for blood purification in sepsis: The Bogotá consensus. Shock.

[10] Hetz H., Berger R., Recknagel P., Steltzer H. (2014). Septic shock secondary to β-hemolytic streptococcus-induced necrotizing fasciitis treated with a novel cytokine adsorption therapy. Int. J. Artif. Organs.

[11] Berkes A., Szikszay E., Kappelmayer J., Kerényi A., Szabó T., Ujhelyi L., Bari K., Balla G., Balla J. (2017). Use of Hemadsorption in a Case of Pediatric Toxic Shock Syndrome. Case Rep. Crit. Care.

[12] Amerson S.J., Hoffman M., Abouzahr F., Ahmad M., Sterling R.K., Gidwani H., Sousse L.E., Dellavolpe J.D. (2024). Sequential Extracorporeal Therapy of Pathogen Removal Followed by Cell-Directed Extracorporeal Therapy in Streptococcal Toxic Shock Syndrome Refractory to Venoarterial Extracorporeal Membrane Oxygenation: A Case Report. Crit. Care Explor..

[13] Miranda G., Neves V., Tjeng R., Lito P. (2025). Hemoadsorption as Adjunctive Therapy in Streptococcal Toxic Shock Syndrome. Cureus.

[14] Broman M.E., Hansson F., Vincent J.L., Bodelsson M. (2019). Endotoxin and cytokine reducing properties of the oXiris membrane in patients with septic shock: A randomized crossover double-blind study. PLoS ONE.

[15] Spaulding A.R., Salgado-Pabón W., Kohler P.L., Horswill A.R., Leung D.Y.M., Schlievert P.M. (2013). Staphylococcal and Streptococcal Superantigen Exotoxins. Clin. Microbiol. Rev..

[16] Carapetis J.R., Jacoby P., Carville K., Ang S.J., Curtis N., Andrews R. (2014). Effectiveness of Clindamycin and Intravenous Immunoglobulin, and Risk of Disease in Contacts, in Invasive Group A Streptococcal Infections. Clin. Infect. Dis..

[17] Evans L., Rhodes A., Alhazzani W., Antonelli M., Coopersmith C.M., French C., Machado F.R., Mcintyre L., Ostermann M., Prescott H.C. (2021). Surviving Sepsis Campaign: International Guidelines for Management of Sepsis and Septic Shock 2021. Crit. Care Med..

[18] Singer M., Deutschman C.S., Seymour C.W., Shankar-Hari M., Annane D., Bauer M., Bellomo R., Bernard G.R., Chiche J.-D., Coopersmith C.M. (2016). The Third International Consensus Definitions for Sepsis and Septic Shock (Sepsis-3). JAMA.

[19] Shankar-Hari M., Phillips G.S., Levy M.L., Seymour C.W., Liu V.X., Deutschman C.S., Angus D.C., Rubenfeld G.D., Singer M. (2016). For the Sepsis Definitions Task Force. Developing a New Definition and Assessing New Clinical Criteria for Septic Shock (Sepsis-3). JAMA.

[20] Zhang L., Srisawat N., Lee C.C., Lee D.H., Lee K., Liu Z., Mohamad Nor F.S., Mustafar R.B., Peerapornratana S., Pham H.M. (2025). Extracorporeal Blood Purification with the oXiris^®^ Filter for Patients with Sepsis and Hyperinflammatory Conditions: The Asia-Pacific oXiris Expert Meeting 2024 Consensus Statements. Blood Purif..

[21] Diab M., Lehmann T., Bothe W., Akhyari P., Platzer S., Wendt D., Deppe A.C., Strauch J., Hagel S., Günther A. (2022). Cytokine Hemoadsorption During Cardiac Surgery Versus Standard Surgical Care for Infective Endocarditis (REMOVE): Results From a Multicenter Randomized Controlled Trial. Circulation.

[22] Rao C., Preissing F., Thielmann M., Wendt D., Haidari Z., Kalisnik J.M., Daake L., Traeger K. (2023). Hemoadsorption Using CytoSorb in Patients with Infective Endocarditis: A German-Based Budget Impact Analysis. J. Cardiovasc. Dev. Dis..

[23] Lei K., Chen A., An X., Guo J., Su B., Li Y. (2025). The use of CytoSorb hemoadsorption in critically ill patients: A narrative review. Front. Med..

[24] Berlot G., Samola V., Barbaresco I., Tomasini A., di Maso V., Bianco F., Gerini U. (2022). Effects of the timing and intensity of treatment on septic shock patients treated with CytoSorb^®^: Clinical experience. Int. J. Artif. Organs.

[25] Wei T., Chen Z., Li P., Tang X., Marshall M.R., Zhang L., Fu P. (2020). Early use of endotoxin absorption by oXiris in abdominal septic shock: A case report. Medicine.

[26] Siew L.Y., Lee Z.Y., Yunos N.M., Atan R., Cove M.E., Lumlertgul N., Srisawat N., Hasan M.S. (2024). Outcomes of extracorporeal blood purification with oXiris^®^ membrane in critically ill patients: A systematic review and meta-analysis. J. Crit. Care.

[27] Kenda S., Gubenšek J., Vovk T. (2026). Antibiotics and Other Drugs Removal by the CytoSorb(^®^) Haemoadsorber: A Systematic Review of Available Evidence. Antibiotics.

[28] Stevens D.L., Bryant A.E. (2017). Necrotizing Soft-Tissue Infections. N. Engl. J. Med..

[29] Gawaziuk J.P., Strazar R., Cristall N., Logsetty S. (2018). Factors predicting health-related quality of life following necrotizing fasciitis. J. Plast. Reconstr. Aesthet. Surg..

[30] Kruppa C., Hutter D.J., Königshausen M., Gessmann J., Schildhauer T.A., Coulibaly M.O. (2019). Necrotizing fasciitis and the midterm outcomes after survival. SAGE Open Med..

